# A case report of adenofibroma arising from the seminal vesicle: A rare giant pelvic mass

**DOI:** 10.3389/fonc.2022.1015499

**Published:** 2022-09-29

**Authors:** Youyi Lu, Di Sun, Jitao Wu

**Affiliations:** ^1^ Department of Urology, The Affiliated Yantai Yuhuangding Hospital of Qingdao University, Yantai, China; ^2^ Department of Pathology, The Affiliated Yantai Yuhuangding Hospital of Qingdao University, Yantai, China

**Keywords:** seminal vesicle, adenofibroma, pelvic mass, case report, tumor, rare

## Abstract

Primary seminal vesicle tumors are extremely rare. Several rare pathological types of primary seminal vesicle tumors have been reported, such as adenocarcinoma, but there is no report on adenofibroma. We report the first case of adenofibroma arising from the seminal vesicle. A 50-year-old man, with no history or clinical evidence of any other tumors, accidentally found a pelvic mass during an ultrasound examination. As the mass grew, the patient developed mild constipation, without genitourinary or other symptoms. All laboratory examinations were normal. MRI of the pelvis revealed a mixed density, measuring 11.7×9.9×8.2cm, well circumscribed mass. The rectum, bladder, prostate and lymph nodes were normal. We successfully performed the open surgery and removed the mass. Histopathological results confirmed that the mass was a primary seminal vesicle adenofibroma. In literature, we found that for biphasic differentiated tumors, it is easy to reduce the accuracy of pathological diagnosis because of insufficient puncture. Therefore, preoperative puncture biopsy for seminal vesicle tumors should be investigated further.

## Introduction

Male pelvic tumors include bladder tumors, prostate tumors, intestinal tumors, and primary malignant lymphoma among others (1). Seminal vesicle tumors are extremely rare in pelvic tumors. The current clinical case reports of seminal vesicle tumors mainly involve secondary tumors from adjacent organs (such as prostate, bladder, rectum or lymphoma), and the common clinical symptoms include urinary tract obstruction, hematospermia, and other non-specific symptoms. Primary seminal vesicle tumors have been rarely reported.

Adenofibroma is common in mammary glands and genital system (uterus, fallopian tubes and ovary) of women, and occasionally in other sites such as the lungs (2, 3) and biliary tract (4). Among men, a few cases have been reported in the testis (5–7), adenofibroma originating from seminal vesicle is an unprecedented report. We report a case of primary seminal vesicle adenofibroma and reviewed the related literature.

## Case presentation

**Figure 1:**
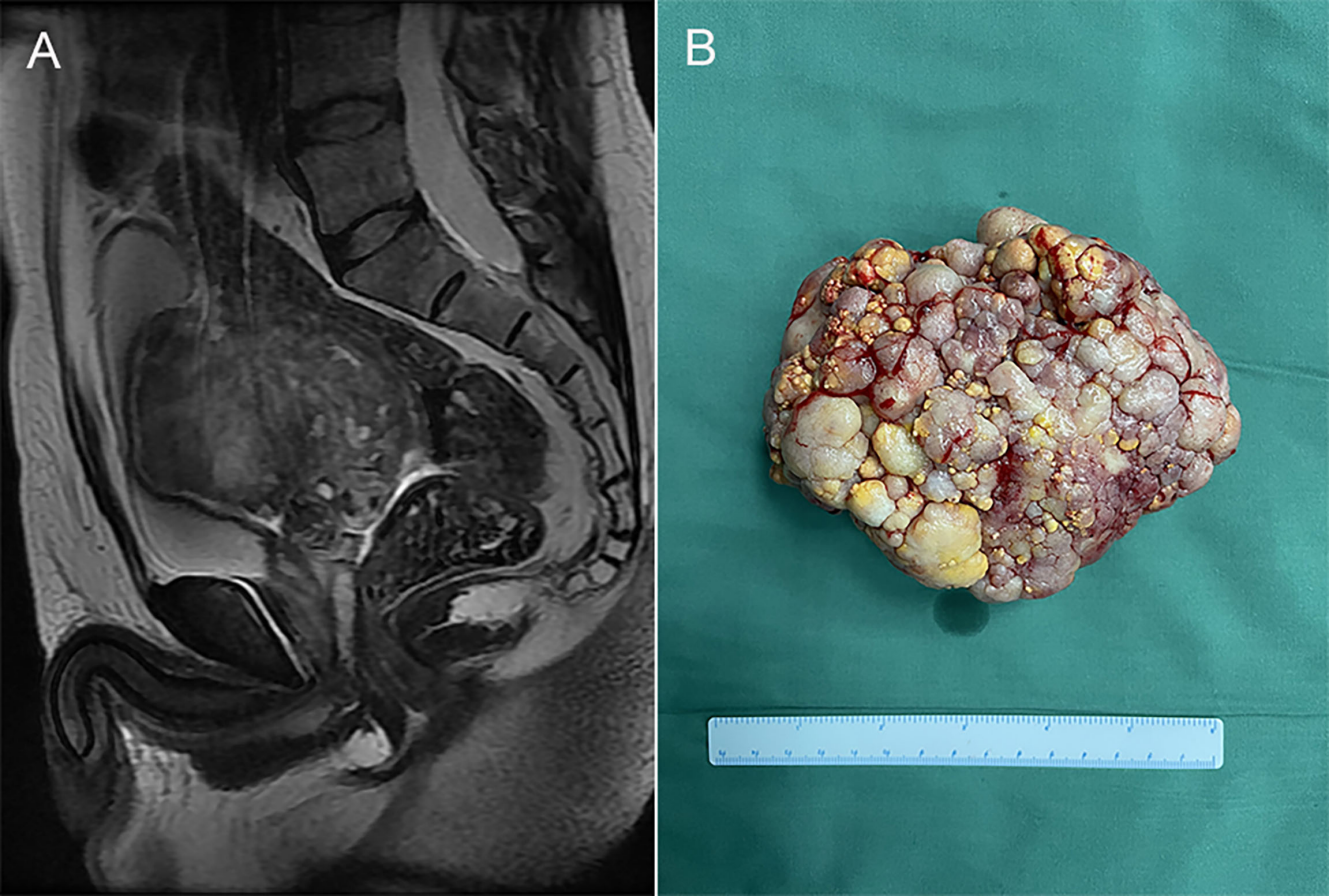
**(A)** Magnetic resonance imaging (MRI) of the pelvis revealed a mixed density, measuring 11.7×9.9×8.2cm, well circumscribed mass. **(B)** The gross specimen exhibited a white-gray, cauliflower-like appearance, uneven with calcification, well encapsulated, measuring 12.5 × 9.5 × 7.0cm.

A 50-year-old man with no history or clinical evidence of any other tumors accidentally found a pelvic mass (4.3×5.0×5.2cm) during an ultrasound examination five years ago. The patient had no clinical symptoms or signs. He chose conservative treatment and regular evaluation of the mass *via* abdominal ultrasound. Eight months ago, the patient was admitted to our hospital because of progressive growth of the pelvic mass. As the mass grew, the patient developed mild constipation, but no genitourinary or other symptoms. Laboratory examinations, including serum tumor markers and free/total prostate specific antigen (PSA) levels were normal. Magnetic resonance imaging (MRI) of the pelvis revealed a mixed density, measuring 11.7 ×9.9×8.2cm, well circumscribed mass (**Figure 1A**). The rectum, bladder, prostate and lymph nodes were normal. Based on findings from imaging examinations, a primary seminal vesicle or gastrointestinal stromal tumor were suspected.

**Figure 2:**
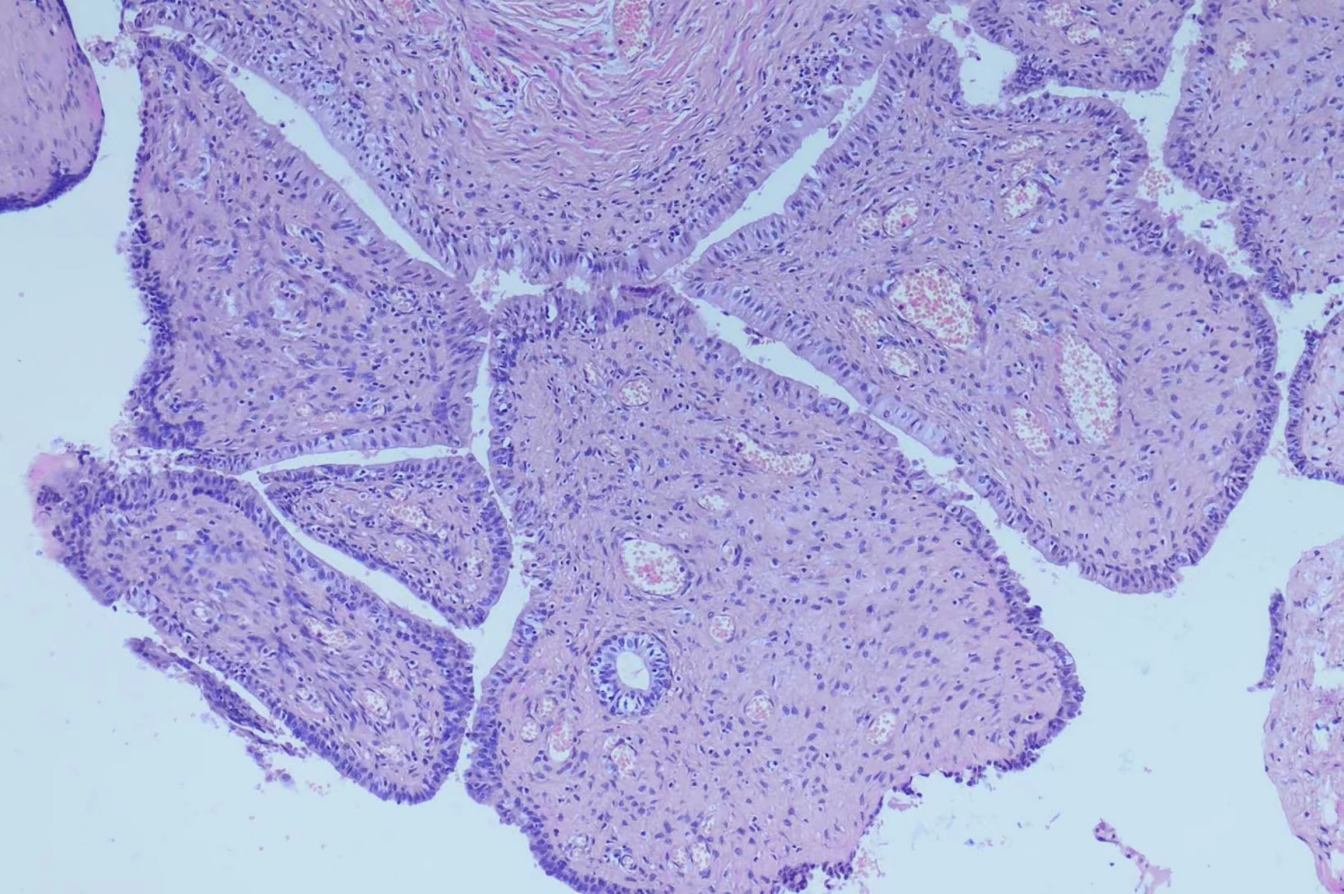
Microscopically, tumor histology revealed fibrous components (mostly) and epithelial components, without atypia. Immunohistochemical results were:CK (+), Vim (+), GATA3 (+), PAX -8(+), CD34 (vessel+), bcl- 2(+), SMA (-), ER (mesenchy me+), PR (mesenchyme+), ki67(+, about 1%).

The patient was subjected to puncture biopsy of the pelvic mass, and the lesion turned out to be mesenchymal tumor arising from the seminal vesicle. Immunohistochemistry showed:CK (glandular epithelium+), CR(-), β-catenin(-), SMA(partial+), CD 34(+), CD117(-), S100(-), Ki67(about 1%+), which did not support gastrointestinal stromal tumor. A definite diagnosis is recommended after complete resection of the tumor. Given the possibility of malignant lesions and other compression symptoms caused by tumor growth, the patient decided to accept complete tumor resection. Laparoscopic exploration revealed a clear boundary between the tumor and surrounding tissues. However, the root of the mass could not be exposed because the mass was so large and took up the whole pelvic cavity. Therefore, we performed open surgery on the patient and successfully removed the mass. The gross specimen exhibited a white-gray, cauliflower-like appearance, uneven with calcification, well encapsulated, measuring 12.5×9.5×7.0cm (**Figure 1B**). No neoplastic invasion of the surrounding tissue was observed and no nodal involvement was found. Microscopically, tumor histology revealed fibrous components (mostly) and epithelial components, without atypia. Immunohistochemical results were:CK (+), Vim (+), GATA3 (+), PAX -8(+), CD34 (vessel+), bcl- 2(+), SMA (-), ER (mesenchyme+), PR (mesenchyme+), ki67(+, about 1%)(**Figure 2**). According to gross specimen and histological morphology, it was unanimously diagnosed by pathologists to be an adenofibroma. The patient recovered well after operation. There was no recurrence or metastasis after an 8-month follow-up.

## Discussion

Primary seminal vesicle tumors are classified as epithelial (adenoma, adenocarcinoma), mesenchymal (leiomyoma, solitary fibrous tumor), mixed epithelial sromal (cystadenoma) and other rare lesions (angiosarcoma, amyloidosis and neuroendocrine carcinoma) (8). Differential diagnosis depends on pathological outcomes. Due to the rarity of primary seminal vesicle tumors, it’s pathological types and clinical practice guidelines have not been well described in textbooks of urology. Some rare pathological types of primary seminal vesicle tumor have been reported, but there are no reports on adenofibroma. We report the first case of primary adenofibroma of the seminal vesicle.

Adenofibroma is a biphasic differentiated tumor. Microscopically, the tumor is a mixed epithelial stromal tumor, mainly composed of benign fibrous components. Although most adenofibromas are benign, there are a few cases of malignant transformation (9). Thus, it is advisable to remove the tumor even if the patient has no symptoms. Long-term patient follow-up after surgery is necessary to identify any recurrence or distant metastasis. Our case provides significant new data for pathological classification of tumors of the seminal vesicle. Pathologists and radiologists should also be aware of this pathological type for differential diagnosis.

Despite its important clinical value, puncture biopsy could not identify the true pathological characteristic features of the tumor in our case. Song et al (10) and Babar (11) also questioned the utility of puncture biopsy in seminal vesicle tumors. Song et al. reported that only 5 of the 12 puncture biopsies were consistent with final pathological diagnosis. And in the study reported by Babar, uncertain biopsy rate of seminal vesicle tumors was as high as 5/17. We considered that for tumors with mesenchymal and epithelial biphasic differentiation, accuracy of pathological diagnosis is significantly reduced because of insufficient puncture. Therefore, preoperative puncture biopsy for seminal vesicle tumors should be investigated further.

## Data availability statement

The raw data supporting the conclusions of this article will be made available by the authors, without undue reservation.

## Ethics statement

Written informed consent was obtained from the individual(s) for the publication of any potentially identifiable images or data included in this article.

## Author contributions

YL and JW were involved in the surgery. Pathological images are provided by DS. YL and DS did literature search. YL drafted the paper. JW revised the paper. All authors have read and approved the final manuscript.

## Funding

This study was funded by Yantai Science and Technology Bureau (2018SFGY117; 2019YD016).

## Conflict of interest

The authors declare that the research was conducted in the absence of any commercial or financial relationships that could be construed as a potential conflict of interest.

## Publisher’s note

All claims expressed in this article are solely those of the authors and do not necessarily represent those of their affiliated organizations, or those of the publisher, the editors and the reviewers. Any product that may be evaluated in this article, or claim that may be made by its manufacturer, is not guaranteed or endorsed by the publisher.

## References

[1] Siegel RL Miller KD Fuchs HE Jemal A. Cancer statistics, 2021. CA Cancer J Clin (2021) 71(1):7–33. doi: 10.3322/caac.21654 33433946

[2] Vitkovski T Zeltsman D Esposito M Morgenstern N. Pulmonary adenofibroma: cytologic and clinicopathologic features of a rare benign primary lung lesion. Diagn Cytopathol (2013) 41(11):991–6. doi: 10.1002/dc.22874 22645035

[3] Wang Y Xiao HL Jia Y Chen JH He Y Tan QY. Pulmonary adenofibroma in a middle-aged man: report of a case. Surg Today (2013) 43(6):690–3. doi: 10.1007/s00595-012-0341-3 23139047

[4] Tsui WM KT L LT C Tse CC. Biliary adenofibroma. a heretofore unrecognized benign biliary tumor of the liver. Am J Surg Pathol (1993) 17(2):186–92. doi: 10.1097/00000478-199302000-00010 8422113

[5] Rogers CG Epstein JI Pavlovich CP. Adenofibroma of the testis discovered at exploration for torsion after trauma. J Urol (2003) 170:1943. doi: 10.1097/01.ju.0000089871.35418.45 14532817

[6] Murao T Tanahashi T. Adenofibroma of the rete testis: a case report with electron microscopy findings. Acta Pathol Jpn (1988) 38:105–12. doi: 10.1111/j.1440-1827.1988.tb01077.x 3364196

[7] Subhawong TK Subhawong AP Hamper UM. Serous adenofibroma of the testicle: case report and review of the literature. J Ultrasound Med (2010) 29(1):135–9. doi: 10.7863/jum.2010.29.1.135 20040787

[8] Dagur G Warren K Suh Y Singh N Khan SA. Detecting diseases of neglected seminal vesicles using imaging modalities: A review of current literature. Int J Reprod BioMed (2016) 14(5):293–302. doi: 10.29252/ijrm.14.5.293 27326413 PMC4910035

[9] Hu W Zhao Y Liu Y Hua Z Liu A. Imaging features of biliary adenofibroma of the liver with malignant transformation: a case report with literature review. BMC Med Imaging (2022) 22(1):47. doi: 10.1186/s12880-022-00775-9 35296268 PMC8928665

[10] Song Y Ning H Yao Z Wu H Shao J Fan M. Primary spindle cell sarcoma of the seminal vesicle: A case report and literature review. Andrologia (2022) 54(4):e14363. doi: 10.1111/and.14363 34984692

[11] Babar M Matloubieh J Macdonald E Cheng J Aboumohamed A Watts K. Diagnosis and treatment of a mixed epithelial stromal tumor of the seminal vesicle: A systematic review. Urology (2022) S0090-42 95(22):00164–9. doi: 10.1016/j.urology.2022.02.012 35231450

